# Medicine and Matrimony

**Published:** 1902-08

**Authors:** 


### *EDITORIAL NOTES AND COMMENTS.*

The Business office of THE JOURNAL-RECORD is No. 64 Marietta Street.

The Editorial office is 318-319-320 The Prudential.

Address all Business communications to Dr. M. B. Hutchins, Mgr.

Make remittances payable to THE ATLANTA JOURNAL-RECORD OF MEDICINE.

On matters pertaining to the Editorial and Original Communications address Dr. Bernard Wolff, Atlanta.

Reprints of original articles will be furnished at cost price. Requests for the same should always be made *on the manuscript.*

We will present, post-paid, on request, to each contributor of an original article, twenty (20) marked copies of THE JOURNAL-RECORD containing such article.

### MEDICINE AND MATRIMONY.

THE following editorial from the *British Medical Journal* is on a most important question and one which is destined to receive more and more serious consideration in the future:

In his address on State Medicine delivered at the annual meeting of the American Medical Association held a few weeks ago at Saratoga Springs, Dr. J. M. Emmert put forward a comprehensive program of reform for the physical betterment of the people of the United States. In that program he includes the medical regulation of marriage. He contends that the State should prohibit the marriage of blood relations up to the second degree, and that all of either sex affected with either congenital or acquired specific or infectious disease, such as venereal or pulmonary affections, confirmed drunkards, criminals, anarchists, and degenerates, should be prevented from entering the estate of matrimony. He insists that every applicant for a marriage license should present a certificate from a medical examiner appointed for the purpose, stating that he is free from any disease that would interfere with procreation or be injurious to offspring.

This is not the first time that the legislative restriction of marriage in the interest of the public health has been before the American Medical Association. It was freely discussed at the annual meeting of that body in 1900, and there was a remarkable unanimity of opinion among those who took part in the discussion in favor of some form of State regulation of marriage. Before that time the Senate of the State of North Dakota had taken the lead in the direction indicated by passing in 1899 the Creed Bill, which provides that licenses to marry are to be given only to such applicants as present a certificate from a board of examining physicians stating that they are free from infectious venereal disease, epilepsy, hereditary insanity, and tuberculosis, and that they are not habitual drunkards. The Tri-State Medical Society of Tennessee, Alabama and Georgia not long ago urged the necessity of legislation for the regulation of marriage in these States. America is a pioneer of sociological experiments, and since then bills similar in scope have been introduced into the Legislatures of Minnesota, Wisconsin, Michigan, Colorado, and several other States. In Europe a bill making it necessary for candidates for matrimony to produce a medical certificate of physical and mental capacity was introduced some time ago into the Bohemian Reichsrath on the initiative of the Bohemian Medical Society. A year or two ago Professor Hegar suggested that the German Parliament should pass a similar enactment, and Professor Pinard lately invited the Académie de Médecine to express an opinion in the same sense. The prohibition of marriage to sufferers from tuberculosis was urged on the attention of governments and statesmen by the Tuberculosis Congress held at Naples in 1899.

All this indicates the beginning of a movement on the part of the medical profession in different countries to induce governments to grapple with what is undoubtedly, from the sociological point of view, a most important question. So far little progress has been made. Statesmen are for the most part profoundly indifferent, and it is hardly to be expected that the public should at this stage take the matter seriously. That it is desirable in the interest of society that the physically and mentally "unfit" should not beget offspring to whom they may transmit their deficiencies is, as an academic proposition, undeniable. But to attempt to secure this desirable end by *force majeure* would be not only tyrannous, but, we believe, futile. Even if to satisfy the sentiment of scientific reformers, laws for the prevention of the marriage of the unfit were passed, it is difficult to see how they could be enforced. You may expel Nature with the fork of the law—*tamen usque recurret.* Love, which is stronger than death, will have an easy victory over the law.

The only practical result of the prohibition of marriage to the diseased and the degenerate would be the increase of concubinage; the birth-rate of the unfit would not be appreciably decreased, and they would have the added brand of illegitimacy to make the struggle for life harder to them. What right has society, for no better end than the physical perfection of the breed, to inflict on persons guilty of nothing but a diseased inheritance, a disability which makes a life overshadowed by ill-health still gloomier?

Doubtless we have the right to protect ourselves and those under our charge against the physical and often moral wreck that follows marriage with a person actually diseased or of unhealthy stock. We should gladly welcome any attempt to deter persons so tainted from inflicting on unsuspecting victims the terrible injury of a union with them. But we deprecate grandmotherly legislation for the preservation of the human species as not only essentially selfish in itself, but as distinctly antisocial in its tendency and probable results.

After all, even with all the assistance for the survival of the unfit given by modern sanitary improvement and humanitarian effort they cannot escape the doom of natural extinction for more than a generation or two. If we are to admit the doctrine that society is justified by the law of self-preservation in purging itself of the unfit, the simpler plan would be to revert to the barbarism of ancient peoples, who solved the problem by destroying them in infancy. This method is at once more effectual and more humane than condemning them to a life of isolation from their kind and exclusion from the chief solace and support against the ills of life to which every human being born into the world has a natural right. In the interest of society itself it might not be altogether wise to drive a large body of unfortunates, who carry in their very constitution the capacity of infinite mischief, into active revolt.

The true mission of the medical profession in this matter is not to promote legislation which is almost certain to defeat its own purpose, but to instruct the public in the dangers, both to the individual and to the community, of unwholesome marriages. The education of the public mind in the practical aspects of a question in which all members not only of the nation and the race, but of the human family, are directly interested would, although the process must needs be slow, make laws for the medical regulation of marriage unnecessary.

